# Antimicrobial Use in Companion Animals: Assessing Veterinarians’ Prescription Patterns through the First National Survey in Chile

**DOI:** 10.3390/ani11020348

**Published:** 2021-01-30

**Authors:** Nicolás Galarce, Gabriel Arriagada, Fernando Sánchez, Vladimir Venegas, Javiera Cornejo, Lisette Lapierre

**Affiliations:** 1 Departamento de Medicina Preventiva Animal, Facultad de Ciencias Veterinarias y Pecuarias, Universidad de Chile, Santiago 8820000, Chile; ngalarce@ug.uchile.cl (N.G.); fernando.sanchez@ug.uchile.cl (F.S.); jacornej@uchile.cl (J.C.); 2 Núcleo Una Salud—FAVET, Facultad de Ciencias Veterinarias y Pecuarias, Universidad de Chile, Santiago 8820000, Chile; 3 Instituto de Ciencias Agroalimentarias, Animales y Ambientales—ICA3, Universidad de O’Higgins, San Fernando 3070000, Chile; gabriel.arriagada@uoh.cl; 4 Escuela de Pregrado, Facultad de Ciencias Veterinarias y Pecuarias, Universidad de Chile, Santiago 8820000, Chile; vnvnege@uchile.cl

**Keywords:** antimicrobial resistance, antimicrobial use, companion animals

## Abstract

**Simple Summary:**

Antimicrobial resistance is a growing global health issue for both animal and public health agencies. One major driver for the development and spread of antimicrobial resistant bacteria is antimicrobial use by animal and health workers. Information about the use of antimicrobials in companion animals has been poorly described worldwide, and currently there is no data at the Latin American or national level, which represents a risk to public health. The aim of this study was to describe the antimicrobial prescription patterns of Chilean companion animal veterinarians by means of a nationally distributed survey. Three hundred twenty-three responses were collected, most of the respondents were female (59.4%). The most frequently reported bacterial diseases were pyoderma (17.2%), followed by otitis (7.4%) and abscesses (7.4%). Critically important or highly important drugs for veterinary and human use were the most frequently used. Only 15% of the veterinarians reported the use of laboratory diagnostic tests prior to prescribing antimicrobials. Our results show that different classes of antimicrobials are used in clinical practices for companion animals without the use of confirmatory laboratory tests, which represents a risk to animal and public health.

**Abstract:**

Although the relationship between the use of antimicrobials and the development of resistant bacteria is well established, information about the use of antimicrobials in companion animals has been poorly described, which represents a risk to public health. The aim of this study was to describe the antimicrobial prescription patterns of Chilean companion animal veterinarians. A nationally distributed survey targeted at companion animal veterinarians was designed. The survey included questions about the veterinarian’s demographics, bacterial diseases treated, prescribed antimicrobials, and the use of laboratory diagnostic tools. Three hundred twenty-three responses were collected, most of the respondents were female (59.4%). The most frequently reported bacterial diseases were pyoderma (17.2%), followed by otitis and abscesses (7.4%). The antimicrobials most used corresponded with critically or highly important drugs for veterinary and human use, including β-lactams (65.3%), quinolones (36.2%) and tetracyclines (23.2%). Only 15% of the veterinarians reported the use of laboratory diagnostic tests, although 67% declared they were aware of the official antimicrobial classification schemes. Our results describe for the first time the usage of antimicrobials by veterinarian practitioners in Chile to treat different diseases in companion animals. The data presented here provide a baseline that could help to promote the implementation of clinical guidelines and regulations in order to improve current treatments.

## 1. Introduction

Antimicrobial resistance (AMR) is a natural defense mechanism that has been present in the environment practically since the appearance of bacteria. However, the evolution and spread of multi-resistant bacteria has increased dramatically in the last 50 years, mainly due to the use of large amounts of antimicrobials in human and veterinary medicine [1,2,3]. AMR represents a major threat to the global economy and public health, where infections caused by antimicrobial resistant bacteria are estimated to cause 10 million deaths per year, and a cumulative cost of US $100 trillion is expected by 2050 if no action is taken to control the spread of AMR [4]. In human medicine, the costs of getting ill from an antimicrobial resistant bacterium have been clearly established [5], but in veterinary medicine, data is limited to productive losses and food health security rather than clinical aspects.

Given the magnitude and global spread of this problem, the World Organization for Animal Health (OIE), the Food and Agriculture Organization of the United Nations (FAO) and the World Health Organization (WHO) have come together to support governments, health care workers, veterinarians, plant professionals and other stakeholders, to focus on AMR under the “One Health” approach, which promotes the responsible use of antimicrobials in humans, animals and plants [6]. Additionally, the WHO and OIE have classified the antimicrobials used in human and veterinary medicine to encourage their proper use [7,8]. However, most of their actions have focused on livestock, with no official policies regarding companion animals such as dogs and cats.

One of the main strategies advocated by the OIE and WHO is the appropriate use of antimicrobials. Several studies have addressed the factors that influence the use of antimicrobials by human healthcare professionals [9,10,11], but studies related to veterinary medicine are scarce. Some authors have suggested that the prescription of antimicrobials by veterinarians is influenced by their perception of the drug’s efficacy, ease of administration, and personal preferences and experiences [12,13,14,15], which is contrary to the recommendations of the OIE. Additionally, veterinarians frequently prescribe critically and highly important drugs for both human and veterinary medicine as the first therapeutic choice [16,17,18]. Moreover, a high proportion of the animals admitted to veterinary hospitals are treated with antimicrobials even when there is no evidence of bacterial infection [19]. These practices certainly represent a risk for animal and public health by increasing the selection pressure of resistant strains. Some international studies have addressed the perception of veterinarians and veterinary students regarding antimicrobial use and have revealed that most had gaps in their knowledge of AMR and the categorization of drugs for antimicrobial therapy, performing susceptibility tests was not common, and there was poor awareness with regard to antimicrobial stewardship and global efforts for the control of this threatening phenomenon [1,20,21].

In Chile, as well as most other countries, actions carried out by official agencies to control AMR have focused almost exclusively on livestock, people and food, thus ignoring companion animal clinical practice. For this reason, the Intersectoral Work Board on AMR in Companion Animals was established in 2018 to focus on AMR in companion animals by strengthening the education of veterinarians and veterinary students on the proper use of antimicrobials and their implications, as well as establishing protocols for antimicrobial usage in pets and facilitating collaboration with official organizations [22]. To date, there are no AMR stewardship programs for any animal species, as well as no published studies of antimicrobial use in companion animal clinical practices in Chile.

Clinical practices for companion animals represent a fundamental area of veterinary medicine, which has increased in relevance in society due to improved ownership practices and technological advances. However, AMR in bacteria isolated from these animals is growing and data on antimicrobial use in cats and dogs is scarce. Thus, the aim of this study was to characterize the antimicrobial use patterns by veterinarians working in companion animal clinical practice in Chile, in order to provide a baseline to establish future regulations and to guide the correct use of these drugs in Chile, and finally, to control the emergence and spread of antimicrobial resistant bacteria.

## 2. Materials and Methods

### 2.1. Study Design

A cross-sectional survey of Chilean companion animal veterinarians was conducted between January and March 2019 to estimate the usage prevalence of different classes of antimicrobials for the treatment of bacterial diseases. A minimum sample size of 300 completed surveys was required to ensure a confidence level of 95% and an absolute accepted error of ±5%. A priori prevalence was set at 75% assuming that the use of antimicrobials in the treatment of infectious diseases in companion animals is very common. In 2019, there were 3091 veterinarians who were declared to be working in companion animal clinical practice as registered in the Chilean Veterinary Medical Association (COLMEVET), so we used this number to adjust for the finite population in the sample size calculation. All of them were invited to participate in the survey via email. The survey was authorized by the Ethics Committee for Research in Human Beings of the Universidad de Chile, and was answered voluntarily by each participant after they signed an informed consent. The survey was available in the online platform SurveyGizmo (Boulder, CO, USA), and consisted of 33 open-ended questions. The survey included animal species that attended the clinical practice, years in practice, awareness of AMR, frequency and the laboratory used for culture and susceptibility testing, common antimicrobials prescribed by disease, effectiveness of antimicrobial treatments, and whether they prescribed a second treatment in the event of the therapeutic failure of the first one. A translated version of the original survey is available in the Supplementary Materials.

### 2.2. Survey Design and Administration

The survey was divided into five modules, where Module 1 characterized the respondent in terms of age, years of professional practice, and specialized studies among others. Module 2 characterized the workplace of the respondents with questions about the location of the clinic/hospital, price range of services, and number of professionals working in the establishment. Module 3 asked about the case histories in recent months, particularly those of bacterial origin. Module 4 collected information about the treatment of bacterial infections named in the previous section, the use of diagnostic tests before prescribing the antimicrobial treatment, effectiveness of the treatment and whether they prescribed a second treatment in the event of therapeutic failure. Module 5 focused on AMR awareness with questions about the list of critical antibiotics established by the WHO and OIE, experiences with diseases with multidrug resistant pathogens, and if there were any protocols concerning the use of antimicrobials in his/her workplace.

This questionnaire was first validated by the Intersectoral Work Board on AMR in Companion Animals. A pre-test was conducted on 10 veterinarians belonging to the Companion Animals’ Medicine Specialist Program of Universidad de Chile. After making the modifications suggested by these experts, the survey was sent to veterinarians by email with a brief explanation of its purpose and the link to the survey The database of the COLMEVET was used, which groups veterinarians throughout the country. No personal information was requested, and all participants completed an informed consent before starting the questionnaire.

### 2.3. Data Management

Results obtained in the SurveyGizmo platform were exported to Excel spreadsheets (Microsoft Corporation, Redmond, WA, USA), where it was reordered in individual tables by modules and the respective answers. Bacterial diseases and antimicrobials used were standardized for further analyses.

### 2.4. Data Analysis

Using the binomial exact method with 95% confidence intervals, the prevalence of prescription antibiotic use by veterinarians was estimated for each antibiotic class reported in the survey. The absolute frequency of each prescribed antibiotic was calculated by choice (first, second or third option), and by disease group; counts were displayed using tables and graphs. Trends between the disease group and good antimicrobial therapeutic practices such as antibiotic susceptibility testing (AST) and/or bacterial culture (BC) were assessed graphically. Associations between good treatment practices and the veterinarian’s years of experience were assessed using independent samples *t*-test. Homogeneity of variance was checked using the Levene’s test. Differences were considered as significant when *p* < 0.05. All statistical analyses and graphs were carried out in Stata v15 (StataCorp, College Station, TX, USA).

## 3. Results

From the total number of surveys sent (*n* = 3091), 323 (10.5%) were fully answered, therefore they were considered suitable for inclusion in the study. All veterinarians who answered the survey work with companion animals, such as dogs and cats, and 59.8% worked in Santiago City, the capital of Chile, while the rest (40.2%) were based in other cities of the country. The average age of the respondents was 35 years old, and 86.4% of them hold additional educational qualifications, such as diplomas, master degrees or PhDs. Additionally, 59.4% of the respondents were women, and 32.2% had been working in the field for one to five years. Table 1 summarizes the results of Modules 1 and 2.

Regarding Module 3, 13.3% of respondents indicated they had not treated any infectious diseases of bacterial origin during the last two months, while 7.1%, 19.3% 23.2%, 9.6% and 40.7% of respondents reported having treated one, two, three, four and five patients with bacterial-based diseases, respectively, in the last two months. A total of 91 bacterial disease entities were reported by the veterinarians; the four most prevalent were pyoderma (17.2%), followed by otitis (7.4%), abscesses (7.4%) and gastroenteritis (4.9%). Table 2 summarizes the results for this module.

**Figure 1:**
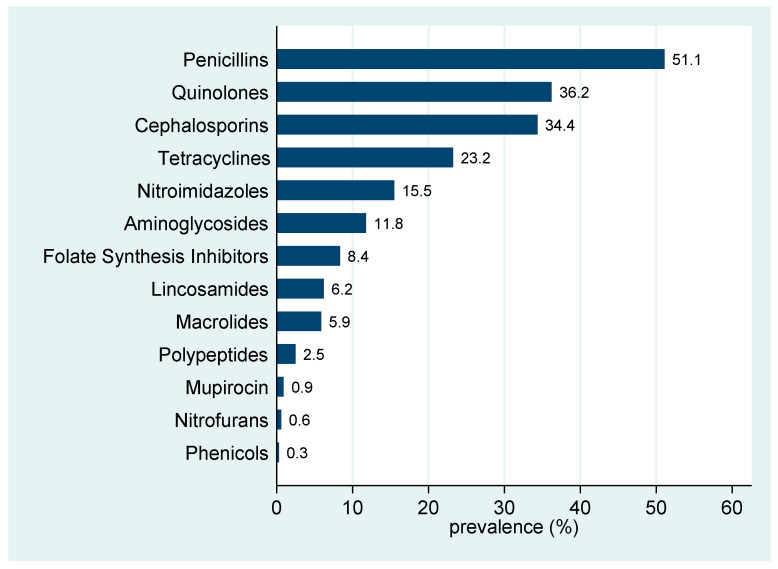
Prevalence of survey respondents who had used antimicrobial agents from each class in the previous 2 months (*n* = 323).

**Figure 2:**
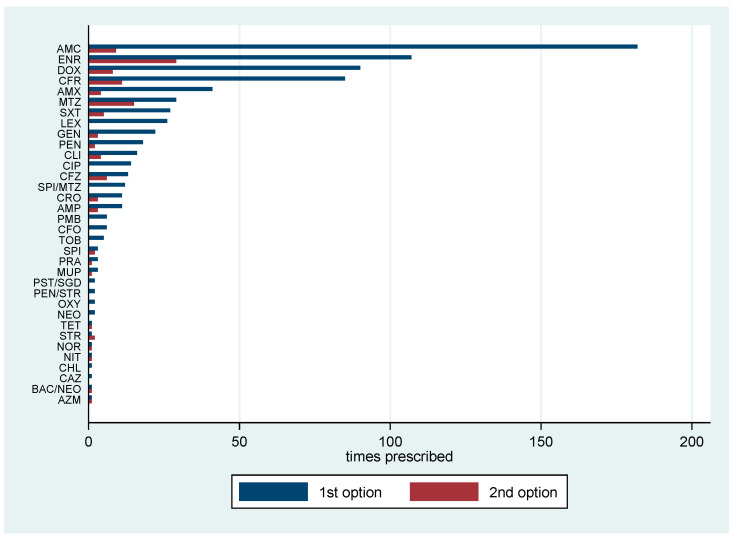
Antibiotics prescribed as first and second treatment options by respondents (*n* = 859). AMC, amoxicillin + clavulanic acid; ENR, enrofloxacin; DOX, doxycycline; CFR, cefadroxil; AMX, amoxicillin; MTZ, metronidazole; SXT, sulfamethoxazole + trimethoprim; LEX, cephalexin; GEN, gentamicin; PEN, penicillin; CLI, clindamycin; CIP, ciprofloxacin; CFZ, cefazolin; SPI/MTZ, spiramycin + metronidazole; CRO, ceftriaxone; AMP, ampicillin; PMB, polymyxin B; CFO, cefovecin; TOB, tobramycin; PRA, pradofloxacin; MUP, mupirocin; SPI, spiramycin; PEN/STR, penicillin + streptomycin; OXY, oxytetracycline; NEO, neomycin; PST/SGD, phthalylsulfathiazole + sulfaguanidine; TET, tetracycline; STR, streptomycin; NOR, norfloxacin; NIT, nitrofurantoin; CHL, chloramphenicol; CAZ, ceftazidime; BAC/NEO, bacitracin + neomycin; AZM, azithromycin.

The most prevalently prescribed antibiotic class was penicillin with 51.1% (95% CI 45.5–56.7) of the veterinarians using antibiotics of this class, followed by quinolones with 36.2% (95% CI 31.0–41.7), cephalosporins with 34.4% (95% CI 29.2–39.8), tetracyclines with 23.2% (95% CI 18.7–28.2), nitroimidazoles with 15.5% (95% CI 11.7–19.9), aminoglycosides with 11.8% (95% CI 8.5–15.8), folate synthesis inhibitors with 8.4% (95% CI 5.6–11.9), lincosamides with 6.2% (95% CI 3.8–9.4), macrolides 5.9% (95% CI 3.6–9.0), polypeptides 2.5% (95% CI 1.1–4.8), mupirocin 0.9% (95% CI 0.2–2.7), nitrofurans 0.6% (95% CI 0.1–2.2) and phenicols with 0.3% (95% CI 0.0–1.7) (Figure 1). The five most frequently prescribed antibiotics as a first choice were amoxicillin+clavulanic acid (182), enrofloxacin (107), doxycycline (90), cefadroxil (85) and amoxicillin (41). As the second option for treatment, the five most frequently prescribed antibiotics were enrofloxacin (29), metronidazole (15), cefadroxil (11), amoxicillin+clavulanic acid (9), and doxycycline (8) (Figure 2).

**Figure 3:**
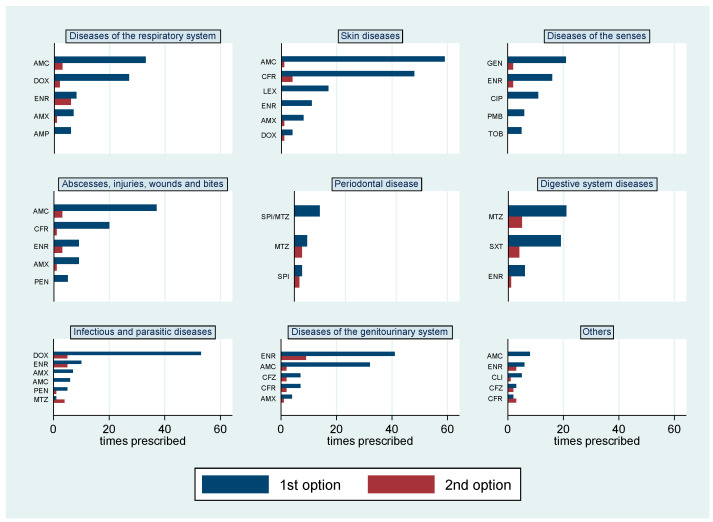
Antibiotics prescribed as first and second treatment options for different diseases reported by respondents (*n* = 853). AMC, amoxicillin + clavulanic acid; DOX, doxycycline; ENR, enrofloxacin; AMX, amoxicillin; AMP, ampicillin; CFR, cefadroxil; LEX, cephalexin; GEN, gentamicin; CIP, ciprofloxacin; PMB, polymyxin B; TOB, tobramycin; PEN, penicillin; SPI/MTZ, spiramycin + metronidazole; MTZ, metronidazole; SPI, spiramycin; SXT, sulfamethoxazole + trimethoprim; CFZ, cefazolin; CLI, clindamycin.

Additionally, 54% of respondents indicated a 100% of efficacy for the first therapeutic option. Amoxicillin+clavulanic acid was the first choice of veterinarians when treating respiratory system diseases, skin diseases, abscesses, injuries, wounds and bites. Gentamicin was the most reported antibiotic used in diseases of the senses, while metronidazole was the first selection for diseases of the digestive system and also in periodontal disease. Both spiramycin and enrofloxacin were reported as the antibiotics first used to treat diseases of the genitourinary system (Figure 3).

**Figure 4:**
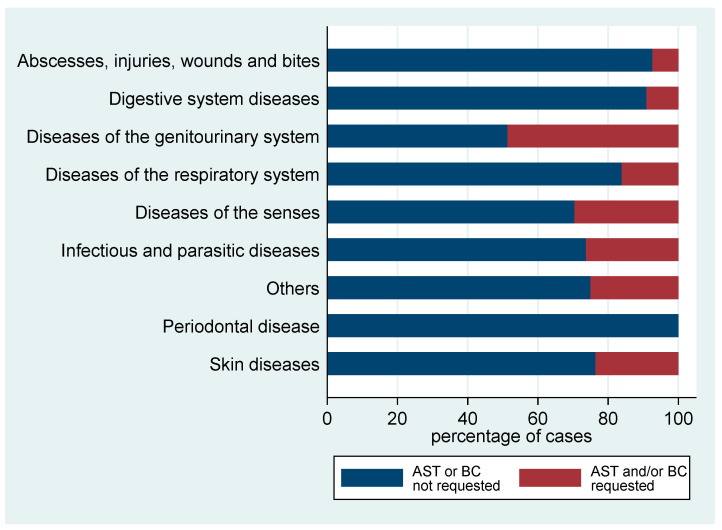
Use of laboratory diagnostic tests for diseases reported and treated by respondents (*n* = 740).

Both BC and AST were requested before beginning antimicrobial therapy in 15.7% of diseases reported by the veterinarians, and in 4.1% of the reported diseases only BC was requested while in 3.6% only AST was conducted. The request for these two procedures was highly correlated (Spearman’s *rho* = 0.76), indicating they are generally requested together. AST and/or BC are more frequently requested by the veterinarian when treating diseases of the genitourinary system (48.6% of cases), diseases of the senses (29.6% of cases), infectious or parasitic diseases (26.3%), other diseases (25%) and skin diseases (23.6%). AST or BC were not requested at all when treating periodontal disease, and rarely for abscesses, injuries, wounds and bites (7.4% of cases) or when treating diseases of the digestive system (9.1% of cases) (Figure 4). An independent samples *t*-test was used to evaluate whether requesting AST or BC is associated with the experience of the veterinarian. The results indicate that there was no significant difference in experience between requesting and non-requesting professionals (*p* = 0.647). The variance in experience between groups was considered as homogenous following a non-significant Levene’s test (*p* = 0.305).

Sixty-seven percent of the respondents indicated having knowledge about the classification of antibiotics according to WHO and OIE, and 26% said they had used those antibiotics for clinical treatment. Additionally, 66% of the surveyed veterinarians stated that there were no protocols in their workplace for the treatment of infectious diseases. Respondents indicated that they obtained the AMR information from laboratory leaflets and from scientific journals.

## 4. Discussion

Antimicrobials constitute an essential tool for the treatment of infectious diseases in humans and animals. However, the improper use of these drugs has contributed to the emergence of AMR. As indicated by the OIE [23], antimicrobial use in companion animal practices is much lower than in farm animals worldwide, but the close relationship between treated animals, veterinary staff, owners and their families, and the community has increased the risk of transfer and infection with antimicrobial resistant pathogens [24]. In Chile, an official report issued in 2019 by the Servicio Agrícola y Ganadero on the sale of antimicrobials used in veterinary medicine revealed that 2283 kg of antimicrobials were sold for use in companion animals, while in production animals, both terrestrial and aquatic, that amount reached 461,933 kg [25]. Only a few countries have policies regarding antimicrobial use in companion animals and data concerning the use patterns of these drugs is scarce [26]. This situation is even more precarious in Asian, African and Latin American countries, which poses a risk for human as well as animal health since antimicrobial resistant bacteria can spread rapidly to diverse hosts and regions. Although there is some awareness about the importance of the use of antimicrobials in the clinical treatment of cats and dogs [26], there have been few investigations on this topic at the global level, and more worryingly, there is no data available in Latin American countries, including Chile. This study is the first report on the use of antimicrobials in companion animals in Chile.

According to our results, most of the respondents were females, ranging in age from 30–39 years old, and working in the Metropolitan Region of Chile. These results are consistent with reports from Chile that indicate that out of 3091 veterinarians, 69.9% (*n* = 1368) are women who work in companion animal clinical practices and most of them work in the Metropolitan Region (46.2%) [27].

One of the survey questions asked for a report on the diseases of bacterial origin diagnosed in the last two months. In general, it was observed that the diagnoses made by the veterinarians were performed without obtaining samples for bacteriological culture, i.e., empirically, so many of the diseases diagnosed do not have a confirmed bacterial etiology. Despite this, we carried out the data analyses with all the diseases indicated by the respondents, since all of them were treated with antibiotics. A wide variety of bacterial diseases treated by veterinarians in companion animals was reported. Of the 91 diseases reported, pyoderma was the most frequently diagnosed. Canine pyoderma is common in small animal practices and frequently leads to the prescription of systemic antimicrobial agents [28]. However, the ability to effectively treat pyoderma, has now been substantially limited by the emergence of multidrug-resistant methicillin-resistant staphylococci [28]. A similar situation has occurred when treating otitis, which was identified as the second most common disease. Otitis externa is the most common ear disease of dogs, with up to 20% of the dog population affected by this disease. Otitis has a multifactorial etiology but is predominantly a bacterial infection [29]. A better understanding of the physiopathology and the risk factors from these and other bacterial diseases, as well as better diagnostic procedures, could reduce the inappropriate use of antimicrobials in animals [28,30]. For this reason, efforts should be focused on developing specific treatment guidelines for each particular disease and species, along with regulations regarding microbial use and surveillance programs for resistance in companion animals, with the aim of improving veterinary use of antimicrobials.

Enhanced penicillins were the primary type of antimicrobial reported in our survey, with amoxicillin + clavulanic acid being the most frequently used antimicrobial (28% of cases). In Australia, a 34% usage of this drug in companion animals was reported [26] and in the UK, amoxicillin + clavulanic was used in 28.5% of cases [31]. Similarly, New Zealand reported a usage of 48% [32] while in Canada, this antimicrobial was reported in 59% of cases [33]. The high level of empirical prescriptions of this β-lactam constitutes a risk for the generation of bacteria that are resistant to β-lactams in pets and humans, especially given that it is considered highly important in human and in veterinary medicine [7,8]. Amoxicillin + clavulanic acid is an antimicrobial widely used in human and veterinary medicine, which is routinely prescribed for respiratory tract infections, skin infections, and urinary tract infections. In the context of the growing global prevalence of multi-resistant strains, international studies have shown that the empirical and unnecessary use of this antimicrobial can select and co-select resistance to aminoglycosides, fluoroquinolones and the production of extended spectrum β-lactamases in gram-negative pathogens [34,35,36,37]. The second most used antimicrobial corresponded to enrofloxacin, followed by cefadroxil, and doxycycline, drugs also considered critically or highly important for both human and veterinary medicine [7,8]. Nevertheless, further investigations are required to determine the volume of use of these antimicrobials and the phenotypic and genotypic susceptibility present in the different pathogens isolated from companion animals in Chile.

When considering the wide diversity of clinical cases reported by those surveyed, veterinarians use the same antibiotics for most of them. This is worrying, since depending on the type and severity of the clinical case, and therefore the response time available to the veterinarian, the clinician should have an arsenal of non-critical antibiotics as a first line of action, and then corroborate their therapeutic effectiveness by conducting laboratory tests. This would contribute to the prudent use of antibiotics without risking the health and welfare of the patient. Additionally, reviewing empirical treatment protocols for each disease may be required in order to use other therapeutic options that meet international criteria for the appropriate use of antimicrobials.

One of the most relevant results of our study is the fact that 85% of the veterinarians carry out empiric treatment of bacterial diseases, while only a small proportion of them (15%) report the use of in vitro susceptibility tests. These results are similar to those reported in Australia and New Zealand, where empiric treatment is the most frequently used [32,38]. According to the Center for Disease Control and Prevention [39], before prescribing antimicrobials, laboratory tests including BC and AST must be performed in order to ensure drug efficacy. This is particularly important in countries with no official data concerning AMR levels. In the few cases in which these tests were requested prior to treatment, we were not able to gather data regarding the methodology used, since laboratories where the tests were performed are private, none of them are supervised by the government, and all of the data is confidential. The use of a standardized methodology, such as that published by CLSI or EUCAST, in which specific breakpoints are used for bacterial pathogens isolated from companion animals, is of critical importance to ensure the trustworthiness of the results of AST [40,41]. The low level of use of microbiological laboratory tests could be influenced by variables that were not analyzed in the present survey. The first is that in Chile, the cost of laboratory tests for companion animals is high and many pet owners are not willing to pay for them. However, here we did not ask about the economic situation of the clients or owners of pets treated by the surveyed veterinarians. Other reasons may be that veterinarians have the perception that laboratory results are unreliable, and that veterinarians only perform microbiological examinations on patients who have recurrent pathologies or where the treatment was not effective. However, these issues were not addressed in the current study, but should be investigated in future surveys to learn why the use of microbiology laboratory is so low in companion animal clinical practice.

Additionally, 54% of veterinarians indicated that 100% of clinical cases were resolved during the first treatment while 20% reported the administration of a second treatment, mostly with enrofloxacin. This resolution rate at first treatment is higher than that reported previously in other countries [42,43] and depends on several factors, such as nosocomial infection, leukopenia, and infection with antimicrobial resistant bacteria, among other. Here, only a low proportion of the respondents perform microbiological analyses, so many of the diagnosis could be mistaken, and therefore their antimicrobial therapy is incorrect. Thus, more efforts must be employed to improve rapid diagnostics to aid empiric therapy by increasing the specificity of the diagnosis, decreasing the time to appropriate therapy and decreasing unnecessary exposure to broad-spectrum antibiotics [44]. The levels of antimicrobial susceptibility in pathogenic bacteria isolated from companion animals remain unclear, and may be important when antimicrobial therapies fail given that several countries do not have surveillance programs for AMR in bacteria isolated from animals. In Chile, there are currently no surveillance programs for AMR in bacteria isolated from animals, and only a few studies have been conducted regarding the susceptibility levels of bacteria isolated from pets. The first study carried out in Chile [45] determined the antimicrobial susceptibility of 52 *E. coli* strains isolated from previously enrofloxacin-treated dogs and cats, and of 18 *E. coli* strains isolated from non-enrofloxacin-treated pets. In the former group, the authors showed that 86.5% of isolates were resistant to enrofloxacin, 82.7% to ciprofloxacin, 80.8% to levofloxacin and moxifloxacin, and 27% to cefpodoxime. Isolates from the control group did not harbor resistant strains to third generation cephalosporins and to fluoroquinolones. Later, [46] determined the susceptibility profiles to different antimicrobials in 48 and 24 strains of coagulase positive *Staphylococcus* isolated from cats with dermal pathologies and from clinically healthy cats, respectively. In addition, the presence of the *mec*A gene, which encodes resistance to methicillin was also determined. The authors found AMR against at least one drug in 75% of the strains of healthy cats and 87.5% of the strains isolated from cats with dermopathy. The *mec*A gene was detected in eight methicillin-resistant strains and also in three sensitive strains. These findings highlight the need to develop AMR surveillance in bacteria isolated from pets, as well as to improve antimicrobial use in Chile.

Another issue identified by this survey, is that a critically important antibiotic is immediately used when the first treatment fails, without evaluating the reason for the failure. In Latin America there are no official guidelines on the use of antimicrobials in companion animals, and there is no data regarding AMR surveillance in these animals. Thus, understanding antimicrobial use patterns is fundamental to develop a baseline for the creation of guidelines and regulations for antimicrobial prescription, and also to promote the use of laboratory analyses, such as BC and AST, in order to reduce the unnecessary prescription of these drugs. This will certainly improve the current incidence of antimicrobial treatments (54%), decrease morbidity and therapy costs, and thereby promote the proper use of these drugs and control the spread of antimicrobial resistant bacteria.

This is the first study to characterize the antimicrobial use patterns of companion animal clinicians in Chile, and as far as we know, in Latin America. In conducting this study, we faced some difficulties that may have biased our research. In this sense, and despite clearly defining our study population as comprising only veterinarians working in companion animal clinics, in Chile there is no formal specialization in any area of veterinary medicine provided by universities or official veterinarian organizations, so veterinarians specialize by obtaining Masters or PhD degrees or by field practice and experience. This situation could have influenced the awareness of veterinarians regarding their clinical practice, which was asked for in our survey. Another limitation was that we did not ask why microbiological laboratory tests were not performed, if the diseases diagnosed in each patient were recurrent, complicated or the specific reasons for therapeutic failures. A third limitation of the study was that we did not investigate the patient medical records. With this data, we could have been assured that the information that the veterinarian may have forgotten when answering the survey would still be recorded and could be analyzed, thus improving the robustness of the results. All these biases should be resolved in future research with a more thorough question design and by conducting face-to-face interviews with the veterinarians.

## 5. Conclusions

This study represents the first survey conducted in Chile and Latin America to describe the use of antimicrobials by veterinarians in dogs and cats. Although this study only included 323 veterinarians, it provided a substantial amount of data that revealed the incidence of bacterial diseases in these animals in Chile, and also the antimicrobial use patterns in their therapy. According to our results, efforts to control AMR development should be focused on performing proper diagnosis and the increased use of microbiological laboratory tests to support diagnosis and appropriate treatment. Furthermore, official efforts are needed to decrease the overall use of antimicrobials in companion animals and increase awareness about correct antimicrobial use, as well as to establish antimicrobial use protocols under the One Health concept.

## Figures and Tables

**Table 1:** Distribution of demographics of companion animal veterinarians (*n* = 323) participating in an online survey to identify antimicrobial use practices in Chile, 2019.

Demographic Factors	Number of Responses (%)
Gender	
Female	192 (59.4)
Male	110 (34.1)
Preferred not to report gender	21 (6.5)
**Age range**	
22–29	65 (20.1)
30–39	170 (52.6)
40–49	66 (20.4)
50–74	22 (6.8)
**Region of Chile**	
Biobío	15 (4.6)
Metropolitana de Santiago	193 (59.8)
Valparaíso	36 (11.1)
Others (13 regions)	79 (24.4)
**Range of working years**	
<1	17 (5.3)
1–5	104 (32.2)
6–10	93 (28.8)
11–15	57 (17.6)
16–20	32 (9.9)
21–25	10 (3.1)
26–40>	10 (3.1)

**Table 2:** Bacterial diseases reported and treated by respondents (*n* = 740).

Diseases	Number of Responses (%)
**Abscesses, injuries, wounds and bites**	**95 (12.8)**
Abscesses	55 (7.4)
Contaminated wounds	18 (2.4)
Others	22 (3)
**Skin diseases**	**157 (21.2)**
Pyoderma	128 (17.2)
Dermatitis	19 (2.6)
Others	10 (1.4)
**Diseases of the senses**	**71 (9.6)**
Conjunctivitis	15 (2)
Otitis	55 (7.4)
Corneal ulcer	1 (0.1)
**Digestive system diseases**	**55 (7.4)**
Gastroenteritis	36 (4.9)
Enteritis	6 (0.8)
Others	13 (1.8)
**Diseases of the genitourinary system**	**111 (15)**
Bacterial cystitis	35 (4.7)
UTI	35 (4.7)
Pyometra	30 (4.1)
Others	11 (1.5)
**Diseases of the respiratory system**	**93 (12.6)**
Pneumonia	29 (3.9)
Infectious tracheobronchitis	27 (3.6)
Others	37 (5)
**Infectious and parasitic diseases**	**95 (12.8)**
Canine ehrlichiosis	21 (2.8)
Mycoplasmosis	21 (2.8)
Others	53 (7.2)
**Periodontal disease**	**27 (3.6)**
**Others**	**36 (4.9)**

## Supplementary Materials

The following are available online at https://www.mdpi.com/2076-2615/11/2/348/s1, File S1: Antimicrobial use patterns survey Companion animals’ version.
