# Supplementary material for: Antimicrobial Use in Companion Animals: Assessing Veterinarians’ Prescription Patterns through the First National Survey in Chile
Source: Animals (Basel). 2021 Jan 30;11(2):348. doi: 10.3390/ani11020348 (PMC7912091; doi:10.3390/ani11020348)
Supplement: Supplementary file 1 [file animals-11-00348-s001.pdf]

Antimicrobial use patterns survey
Companion animals' version

Welcome:
Dear participant:
The Faculty of Veterinary and Livestock Sciences of the University of Chile thanks you for your time and willingness to answer the following survey.
In it you will find 5 modules with different types of questions and it has an average duration of approximately 15 minutes.
The information provided is compiled for exclusively investigative purposes, being totally confidential, not being manipulated by third parties to the participants of this research or released for commercial use.
There are NO good or bad answers, ALL answers are important for the study to be carried out.
Your participation is completely voluntary.
For any question or suggestion, contact: encuesta-antibioticos@veterinaria.uchile.cl
Thank you for your participation!

-Are you a veterinarian?
-Informed consent *
-I understand that my participation is voluntary and anonymous.
-I am over 18 years of age and I agree to participate in this study having been informed of its purpose and principles.

Module 1: Characterization of the respondent.

1- How old are you?
2- You are male or female?
3- Are you dedicated to a specific area or specialty at companion animal's clinical practice? Which?
4- Have you completed additional studies to complement your professional training within the last 10 years?
Indicate which (you can select more than one alternative)
Diploma
Magister
Doctorate/PhD
Other
5- How many years have you been working in companion animal's clinical practice?
6- During the time that you have carried out your current work, has this been your only job or has had income from other work activities (sale of products, sporadic personal attention, etc.) even when are these minor? *
-I've had income from only one job
-I have had income from other work activities
7- Considering an average month, how many hours do you work per week in your main activity? * Think of "main activity" as your most time-consuming job in the month.
-Less than 30 hours (half a day)

-31 to 44 hours
-45 hours
-46 or more hours

8- How long have you been working in your main activity?
-Less than 1 year
-Between 1 to 3 years
-Between 3 to 5 years
-Between 5 to 10 years
-More than 10 years

Module 2: Characterization of the respondent's work place.

9- In which commune is it located?

10- How many veterinarians work in the clinic where you work?

11- Considering an average week, how many animals does the clinic normally attend?

12- What is the average value of a medical attention? (Chilean pesos)
-Less than $ 5,000
-Between $ 5,000- $ 10,000
-Between $ 10,001- $ 15,000
-Between $ 15,001- $ 20,000
-More than $ 20,000

Module 3: Diseases in companion animal's clinical practice.

13- How many diseases of bacterial origin have you treated in the last two months?
-1
-2
-3
-4
-5
-None

14- What are the bacterial diseases that you have treated in the last two months? Name them.

Module 4: About the diseases.

15- How many antibiotics did you use in the first treatment? (Answer for each one of the indicated bacterial diseases)
-1
-2
-3
-None

16- What antibiotics did you use? (Answer for each one of the indicated bacterial diseases)

17- Were laboratory tests done before prescribing these antibiotics? (Answer for each one of the indicated bacterial diseases)
-Yes
-No

18- If so, what tests do you performed? (You can select more than one alternative) (Answer for each one of the indicated bacterial diseases)

Biochemical profile
Hemogram
Antibiogram
Bacterial culture
Other

19- How often are these tests performed before prescribing these antibiotics? (Answer for each one of the indicated bacterial diseases)
-Always
-Usually
-Occasionally
-Never

20- In what type of laboratory do you perform these tests? (Answer for each one of the indicated bacterial diseases)
-University laboratory
-Private laboratory
-Inside the clinic
-Other

21- For each declared antibiotic specify: (Answer for each one of the indicated bacterial diseases)
-Extra-label use? (yes/not)
-Effectiveness (%) (Remission of clinical signs post-treatment)

22- Did you prescribe a second treatment? (Answer for each one of the indicated bacterial diseases)
-Yes
-No

23- If you changed your antibiotic in the second treatment, which was the criteria that guide your decision? (You can select more than one alternative) (Answer for each one of the indicated bacterial diseases)
-Ineffectiveness
-Adverse effects
-Costs
-Laboratory test results
-Diagnostic failure
-Other

Module 5: Regarding the use of antibiotics.

24- If you have any questions regarding the use of a certain antibiotic, where do you look for information? (You can select more than one alternative)
-Web
-Scientific journals
-Prospects / Laboratories
-Direct consultation with SAG
-Colleagues in the area
-Other: name it
-Does not seek information

25- Are you aware of the list of critical antibiotics established by the WHO and/or the OIE?
-Yes
-No
26- How did you find out about these list(s)?
-University
-Job
-Seminars
-Internet
-Other: name it
27- Have you faced an infectious agent from the highest risk list?
-Yes
-No
28- Of the previously declared diseases, in which of them did you prescribe highest priority antibiotics?
29- Have you had to resort to the use of top-priority antibiotics?
-Yes
-No
30- For the same patient, have you had to use more than one top priority antibiotic? *
-Yes
-No
31- At the level of the clinic where you work, is there a written protocol on the use of antibiotics against certain diseases?
-Yes
-No
-I do not know
-Is in development
32- If there is a written protocol, do you consider that it is actually used?
-Always
-Usually
-Occasionally
-Never
-I do not know
33- If there is a protocol, what are the sources of information used for its creation? (You can select more than one alternative)
-Empirical knowledge
-SAG
-Scientific websites and / or journals
-Experiences of other colleagues
-Seminars or specialized congresses
-Books
-Others
